# Effect of Closure of Anterior Abdominal Wall Layers on Early Postoperative Findings at Cesarean Section: A Prospective Cross-sectional Study

**DOI:** 10.1055/s-0041-1726057

**Published:** 2021-03-30

**Authors:** Ersin Çintesun, Ayşe Gül Kebapçılar, Mustafa Gazi Uçar, Setenay Arzu Yılmaz, Mete Bertizlioğlu, Çetin ÇELİK, Özlem Seçilmiş Kerimoğlu

**Affiliations:** 1 Department of Obstetrics and Gynecology, Selçuk University Medicine Faculty, Selçuklu, Konya, Turkey

**Keywords:** analgesia, cesarean, intestinal motility, parietal peritoneum, rectus abdominis muscle

## Abstract

**Objective** To investigate the effect of closure types of the anterior abdominal wall layers in cesarean section (CS) surgery on early postoperative findings.

**Methods** The present study was designed as a prospective cross-sectional study and was conducted at a university hospital between October 2018 and February 2019. A total of 180 patients who underwent CS for various reasons were enrolled in the study. Each patient was randomly assigned to one of three groups: Both parietal peritoneum and rectus abdominis muscle left open (group 1), parietal peritoneum closure only (group 2), and closure of the parietal peritoneum and reapproximation of rectus muscle (group 3). All patients were compared in terms of postoperative pain scores (while lying down and during mobilization), analgesia requirement, and return of bowel motility.

**Results** The postoperative pain scores were similar at the 2 ^nd^ , 6 ^th^ , 12 ^th^ , and 18 ^th^ hours while lying down. During mobilization, the postoperative pain scores at 6 and 12 hours were significantly higher in group 2 than in group 3. Diclofenac use was significantly higher in patients in group 1 than in those in group 2. Meperidine requirements were similar among the groups. There was no difference between the groups' first flatus and stool passage times.

**Conclusion** In the group with only parietal peritoneum closure, the pain scores at the 6 ^th^ and 12 ^th^ hours were higher. Rectus abdominis muscle reapproximations were found not to increase the pain score. The closure of the anterior abdominal wall had no effect on the return of bowel motility.

## Introduction

The attainment of safe delivery for both mother and baby via cesarean section (CS) is one of the important achievements of modern obstetrics. Cesarean section is one of the most frequently performed operations in the world, and its frequency is increasing. 1 2 Cesarean section is defined as the delivery of the fetus by abdominal and uterine incision. As with every surgical procedure, there are many variations in carrying out CS, and the operation varies among surgeons. All stages of CS can be performed with different techniques. The effort to compare the different procedures of CS and to find a standard method of surgery has been ongoing for a long time. 3 4 5

There are various studies comparing outcomes of closure and non-closure of the parietal and visceral peritoneum, and rectus muscle reapproximation and no approximation. In these studies, postoperative pain, need for analgesia, adhesion, infection, fever, endometritis, and duration of hospitalization were examined, 5 6 7 8 9 and different results were obtained. Studies that compared postoperative pain and need for analgesia focused on the reapproximation of the rectus muscle without comparing parietal peritoneum closure or non-closure. 8 9 In addition, there is no study investigating the relationship between the postoperative return of bowel motility and closure of the anterior abdominal wall layers.

In this study, we investigated the effect of closure types of the anterior abdominal wall layers on postoperative pain, need for analgesia, and postoperative return of bowel motility.

## Methods

The present study was designed as a prospective cross-sectional study between October 2018 and February 2019 in patients undergoing their CS at a university hospital. This study was conducted on patients who had standard CS surgical procedures in our clinic and who were eligible for the study. Anterior abdominal wall closure patterns vary from surgeon to surgeon in our clinic. This stage is implemented in three different ways. Group assignments were made by browsing the operation notes. Each patient was randomly assigned to one of these three groups:

**Group 1:** Both the parietal peritoneum and rectus abdominis muscle were left open;
**Group 2:** Parietal peritoneum closure only;
**Group 3:** Closure of the parietal peritoneum and re-approximation of rectus muscle.

The inclusion criteria were being at 37 or more weeks of gestation, not having systemic disease, having standard CS procedures under spinal anesthesia. Elective CS patients and patients in labor were included the study. The exclusion criteria included the following: patients with preterm births (less than 37 gestational weeks); administration of anesthesia besides general anesthesia; fetal distress; maternal hypertensive disorders; maternal systemic diseases; preeclampsia; premature rupture of membranes; chorioamnionitis; pregestational and gestational diabetes; placental invasion anomalies; coagulation disorders; multiple pregnancies; renal, liver, and gastrointestinal disease; history of > 3 previous CS; presence of dense adhesions; and psychiatric disorders.

Approval from the institutional local ethics committee was obtained, and each patient provided a signed informed consent for their participation in the study. All staff members (nurses and investigators) were blinded to the study groups, and all postoperative procedures were made while remaining blinded. The health care provider who questioned the patient about pain after the operation and medicated the patient did not know which procedure was applied.

### Postoperative Patient Follow-up

As a standard treatment in our clinic, we use intramuscular diclofenac for analgesia for the first 24 hours (1 g every 8 h) and orally 3 times per day thereafter. In the case of patients who describe pain (visual analogue scale [VAS] ≥ 30) in spite of the treatment with diclofenac, we administer narcotics as an analgesic instead of diclofenac. All patients receive preoperative prophylactic intravenous antibiotics (cefazolin 2 g). Liquid food intake and mobilization of the patients are performed at the 6th postoperative hour. The nursing staff monitor and record maternal blood pressure, pulse activity, and urinary output hourly during the first 24 hours.

In the present study, postoperative pain was measured using a VAS (0 = no pain and 100 = worst pain ever). All mothers completed the VAS score at the 2 ^nd^ , 6 ^th^ , 12 ^th^ , and 18 ^th^ hours after surgery with the assistance of nursing staff. Pain scores were recorded at the 2 ^nd^ hour while lying down, and at other hours, pain scores were recorded both while the patient was lying down and during mobilization. The time to return of bowel function was recorded from the end of the surgery to the times of the first passage of flatus and stool. No medical or mechanical intervention was performed for passing flatus and stool. The patients were discharged at 48 hours postoperatively. No intervention was used for patients who did not pass stool within 48 hours.

### Surgical Technique

Cesarean section is performed in our clinic as described below. All CSs are performed by the senior assistant. Nearly all surgeries are performed under spinal anesthesia. A standard technique is used in all procedures. All women have a Pfannenstiel-type transverse incision. The subcutaneous tissue layer is dissected using the fingers, and then a small transverse incision is made, medially, with a scalpel and extended laterally using scissors in the fascial layer. The rectus muscles are separated bluntly. The peritoneum is opened with the forefinger. A bladder flap is formed, and a low transverse incision is made in the uterus. The uterine incision is closed using a single-layer continuous locked suture with a Vicryl 1.0 suture (Ethicon, Johnson & Johnson, Cincinnati, OH, USA). The abdominal cavity is cleaned from amniotic fluid and blood. The closure of the parietal peritoneum and rectus muscle in our clinic varies according to the preference and experience of the surgeon. The parietal peritoneum is closed using a continuous Vicryl 2.0 suture (Ethicon Johnson & Johnson). The rectus muscles ere reapproximated using three loose vertical midline interrupted sutures with Vicryl 2.0 sutures (Ethicon Johnson & Johnson). Sutures are placed about 1 cm from the edge of the incision and 1 cm apart, without excessive tension. Subcutaneous fat is closed when the tissue is thicker than 2 cm. Skin is reapproximated using a continuous subcuticular suture with 2.0 polypropylene (Ethicon Johnson & Johnson). All operative procedures are performed by the same surgeon. The day of CS is considered as day 0.

### Statistical Analyses

The G*Power 3.0.10 software (Franz Faul, Uni Kiel, Germany) was used for calculating the sample size. 10 Sample size was calculated with an alpha of 0.05, a power of 80, and medium effect size (f) 0.25. Given this calculation, the minimum required sample size was 53 patients in each group. One hundred and eighty patients were included in the study. Statistical analyses were performed using the IBM SPSS Statistics for Windows 21.0 software (IBM Corp., Armonk, NY, USA). Parametric continuous data were presented as means ± standard deviation, nonparametric continuous data were presented as medians (min–max). Categorical variables were expressed as numbers (percentages). For statistical analysis, one-way analysis of variance (ANOVA), Kruskal-Wallis, Mann-Whitney U, and Pearson chi-squared tests were used, as appropriate; *p* < 0.05 was considered significant.

## Results

A total of 180 women who underwent CS for various reasons, including planned repeated cesarean delivery with up to maximum three previous CSs, were enrolled in the study. All patients were similar in groups with respect to age, weight, and body mass index (BMI).

Sixty women were allocated to each group. The most frequent indications for CS were as follows: elective repeat CS, malpresentation, and macrosomia. The demographic and physical conditions of the subgroups are summarized in table 1 . Age, gravidity, parity, weight (before CS), weight (before pregnancy), weight gain during pregnancy, height, and BMI (before pregnancy) were not significantly different between the groups. The BMI (before birth) was significantly higher in group 2 than in groups 1 and 3.

**Table 1:** Comparison of demographic characteristics and physical conditions

	Group 1 N = 60	Group 2 N = 60	Group 3 N = 60	*p* -values (among groups)
Age*	28 ± 6.1	30 ± 5.7	29 ± 5.7	0.495
Gravidity**	2 (1–6)	2 (1–6)	2 (1–8)	0.380
Parity**	1 (0–4)	1 (0–4)	1 (0–5)	0.782
Weight (before Caesarean section)*	78 ± 13	81 ± 14	77 ± 13	0.124
Weight (before pregnancy)**	65 (40–115)	68 (43–103)	63 (47–105)	0.177
Height*	162.4 ± 6.2	162.4 ± 5.8	162.6 ± 5.7	0.982
Weight gain during pregnancy**	10 (2–20)	11 (3–28)	11 (2–21)	0.124
BMI (before pregnancy)**	24.8 (18–40)	26.4 (16–38)	23.4 (18–38)	0.108
BMI (before birth)**	28.6 (21–40) a	30 (18–40) a b	28 (20–42) b	0.030

*One-way analysis of variance (*) and Kruskal-Wallis (**) variance analysis as appropriate.

Bold values represent *p* < 0.05.

Data shown mean ± SD and median (minimum-maximum).

a P1< 0.05 versus P2 (Kruskal-Wallis, Mann-Whitney U-test).

b P2 < 0.05 versus P3 (Kruskal-Wallis, Mann- Whitney U-test).

**Fig. 1:**
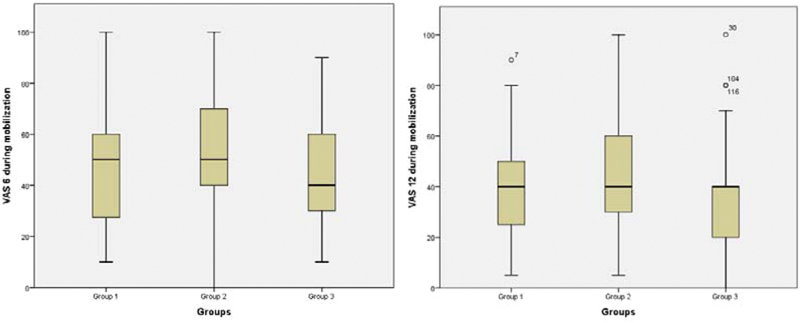
Boxplot graphics of postoperative pain scores at the 6th and 12th hours during mobilization.

The comparison of the groups in terms of the VAS of the subgroups is summarized in Table 2 and Figure 1 . At the 2 ^nd^ postoperative hour, the VAS was evaluated only while lying down, whereas at the other times, it was evaluated while lying down and during mobilization. The postoperative pain scores were similar at the 2 ^nd^ , 6 ^th^ , 12 ^th^ , and 18 ^th^ hours while lying down. During mobilization, the postoperative pain scores were different between the groups at the 6 ^th^ and 12 ^th^ hours, and the pain scores at the 6 ^th^ and 12 ^th^ hours were significantly higher in group 2 than in group 3.

**Table 2:** Comparison of groups in terms of visual analog scale (VAS)

	Group 1 N = 60	Group 2 N = 60	Group 3 N = 60	*p* -values (among groups)
VAS 2nd hour	60 (10–100)	70 (10–100)	60 (10–95)	0.077
VAS 6th hour (lying down)	30 (0–90)	30 (0–70)	27.5 (0–80)	0.203
VAS 6th hour (mobilized)	50 (10–100)	50 (10–100) b	40 (10–90) b	0.029
VAS 12th hour (lying down)	20 (0–85)	27.5 (0–90)	20 (0–90)	0.201
VAS 12th hour (mobilized)	40 (5–90)	40 (5–100) b	40 (0–100) b	0.022
VAS 18th hour (lying down)	10 (0–40)	20 (0–50)	20 (0–50)	0.157
VAS 18th (mobilized)	27.5 (0–70)	30 (10–90)	30 (0–70)	0.188

Kruskal-Wallis (Mann-Whitney U-test) variance analysis as appropriate.

Bold values represent *p* < 0.05.

Data shown median (minimum–maximum).

b P2 < 0.05 versus P3.

The comparison of the groups in terms of analgesia requirements is summarized in Table 3 . The use of diclofenac was significantly different between the groups, being higher among patients in group 1. Meperidine requirements were similar in all groups. The use of diclofenac in group 1 was found higher than in group 2. The first gas and stool expulsion times for each group are summarized in Table 4 . There was no difference between the groups' first flatus and stool passage times.

**Table 3:** Comparison of groups in terms of analgesia requirements

	Group 1 N = 60	Group 2 N = 60	Group 3 N = 60	*p* -values (among groups)
Diclofenac	1 (0–2) ^a^	1 (0–2) ^a^	1 (0–4)	0.043
Meperidine	1 (0–1)	1 (0–2)	1 (0–1)	0.177

Kruskal-Wallis variance analysis as appropriate; Bold values represent *p* < 0.05; Data shown median (minimum-maximum); ^a^ P1 < 0.05 versus P2 (Kruskal-Wallis, Mann-Whitney U-test)

**Table 4:** Comparison of groups in terms of first passage of flatus and stool times

		Group 1 N = 60	Group 2 N = 60	Group 3 N = 60	*p* -values (among groups)	
Time to first pass flatus			16 (4–33)	16 (1–40)	14.5(2–41)	0.626
	Before 24h	47 (31.8%)	52 (35.1%)	49 (33.1%)	0.486
	24th hour and later		13 (40.6%)	8 (25.0%)		11 (34.4%)
Time to first pass stool	Before 24h	8 (27.6%)	13 (44.8%)	8 (27.6%)	0.265
	24–48 hours		29 (31.2%)	27 (29.0%)		37 (39.8%)
	48th hour and later		23 (39.7%)	20 (34.5%)		15 (25.9%)

Kruskal-Wallis and Pearson chi-Squared test; Data shown number (%) and median (minimum-maximum).

## Discussion

The frequency of cesarean section is steadily increasing all over the world, as it is in Turkey. 1 2 11 The CS rate, which was 14.3% in 1993, increased to 51.9% in 2013 in Turkey. 12 There is no standard method for CS in the world. There are many procedural differences, from the opening to the closure of the skin incision. 13 14 15 16 We investigated the effect of the closure types of the anterior abdominal wall layers on early postoperative findings of bowel motility in this study. Postoperative pain scores at 6 and 12 hours were significantly higher in group 2 than in group 3 during mobilization. Diclofenac use was significantly higher in patients in group 1 than in group 2. Meperidine requirements were similar between the groups. There was no difference between the groups in terms of first flatus and stool passage times.

When searching the literature, many studies comparing different stages of CS from skin incision to uterine closure 4 7 8 14 17 were found. For example, Palatnik and Grobman 18 found that vertical incisions did not improve maternal and fetal outcomes, and Daykan et al. 19 found that skin closure using glue or a monofilament synthetic suture had similar results. With consideration to the closure of the uterus, Marceau et al. found that two-layer closure was more effective than single-layer closure. 17 There are also studies suggesting that the risk of uterine dehiscence and rupture has not changed. 20 In peritoneal closure, Kapustian et al. 7 found that closure of the parietal peritoneum did not change the adhesion ratio. 7 However, there are some studies showing that non-closure of the peritoneum is associated with increased adhesions. 5 21

There has been a long-standing hypothesis that there is a relationship between the approximation of the rectus abdominis muscle and pain, 3 22 but, finally, two randomized controlled trials were recently published 8 9 ; Lyell et al. 8 found that rectus muscle reapproximation increased immediate postoperative pain without differences in operative time, surgical complications, or maternal satisfaction, and Omran et al. 9 found that rectus muscle reapproximation among women undergoing primary CS was associated with a significant increase in postoperative pain and analgesic requirements. Both studies, however, focused on rectus muscle approximation versus no approximation and did not examine its relationship with the closure and non-closure of the parietal peritoneum. In our study, closure of the abdominal wall layers was examined as three separate groups. Postoperative pain scores were found to be similar at the 2 ^nd^ , 6 ^th^ , 12 ^th^ , and 18 ^th^ hours while the patients were lying down. During mobilization, postoperative pain scores were different between the groups at 6 and 12 hours. Pain scores at 6 and 12 hours were found to be significantly higher in the parietal peritoneum closure group than in the group undergoing both closure of the parietal peritoneum and reapproximation of the rectus muscle. The approximation of the rectus muscle did not increase postoperative pain and analgesia requirement.

Postoperative intestinal dysfunction is a physiologic response to abdominal surgery and is more common in open surgery, such as CS. 11 Gastrointestinal dysfunction may cause abdominal discomfort due to the accumulation of intestinal contents following CS and may cause a wide range of symptoms, such as nausea, vomiting, or abdominal pain. For this reason, postoperative hospital stay may be prolonged. 2 23 Few studies in the literature have examined the return of intestinal motility after CS. The reason why some patients have early or late bowel motility and its relationship to surgical technique has not been investigated. Studies examining the effect of gum chewing and early oral feeding are available in the literature. 24 25 The aim of this study was to investigate the effect of a tighter closure of abdominal compartment on intestinal motility after closure or non-closure of the rectus muscle or peritoneum. We found that closure of the anterior abdominal wall had no effect on bowel motility.

This prospective cross-sectional study ensured standardization of factors that could affect postoperative pain. All women participating in our study received the same intraoperative anesthesia, followed the same postoperative pain management protocol, and underwent similar surgical techniques so as to reduce the number of potentially confounding variables. Accordingly, we excluded patients with conditions that might influence pain and bowel motility.

The limitations of this study include the follow-up of only postoperative short-term results; the long-term results of aspects such as uterine involution, adhesions, and diastasis recti were not studied. Also, the lack of questioning the patients' previous pain conditions, having no data about the duration of surgery, blood loss, and episodes of nausea and vomiting were other limitations. In addition, mobilization times that could potentially affect gastrointestinal motility could not be controlled. The strength of this study is that it is the first to investigate the effect of closure of the anterior abdominal wall on the postoperative return of bowel motility.

## Conclusion

In the group with only parietal peritoneum closure, the pain scores at the 6th and 12th hours were higher. Rectus abdominis muscle reapproximations were found to not increase the pain score. The closure of the anterior abdominal wall had no effect on the return of bowel motility. Whether or not the anterior abdominal wall is closed has no effect on bowel motility and analgesia requirement. The studies that consider analyzing directly intraabdominal pressures, intestinal motility time, mobilization time of the patient and measuring pain scores more objectively can give more consistent results.
